# Social Acceptance of Aquaculture in Andalusian Atlantic Coast (Spain): An Emerging Economy Sector

**DOI:** 10.3390/foods9070910

**Published:** 2020-07-10

**Authors:** José Ruiz-Chico, Antonio R. Peña-Sánchez, José M. Biedma-Ferrer, Mercedes Jiménez-García

**Affiliations:** 1 INDESS (University Institute for Sustainable Social Development), University of Cadiz, 11406 Jerez de la Frontera, Spain; rafael.pena@uca.es (A.R.P.-S.); josemaria.biedma@uca.es (J.M.B.-F.); mercedes.jimenezgarcia@uca.es (M.J.-G.); 2 Department of General Economy, Faculty of Social Sciences and Communication. University of Cadiz, Avda. de la Universidad, 4-11406 Jerez de la Frontera, Spain; 3 Department of Business Administration, Faculty of Social Sciences and Communication. University of Cadiz, Avda. de la Universidad, 4-11406 Jerez de la Frontera, Spain

**Keywords:** carrying capacity, consumer satisfaction, economics, employment, environment, fish, food, quality, sustainability

## Abstract

Aquaculture is an important source of food. This document analyses its acceptance by consumers from the perspective of social carrying capacity. This concept determines the point at which its development begins to be excessive, and poses difficulties to its geographical area. In this context, our work is innovative, since, although Spain is the leading aquaculture producer in the European Union, not much research on this aspect has been developed. With this objective, we carried out 579 personal interviews with consumers from the Andalusian Atlantic coast (Spain), to determine an index of acceptance for this food and its industry. After applying a principal component analysis, we stratified the samples following Dalenius-Hodges method. According to our surveys, there is a high level of acceptance, which would place aquaculture far from its saturation point, the level at which this sector generates relevant problems for society in terms of economy or environment. Respondents also recommended its further development. Women and low-income consumers were the groups with the greatest reticence about this sector. We consider that general acceptance would be improved by implementing informative campaigns, especially for these groups, that would extend consumers’ knowledge and improve their perception of this kind of food.

## 1. Introduction

Nowadays, aquaculture is an important source of aquatic products [1], being one of the fastest- growing food producers [2,3,4]. We have been able to witness a revolution in this sector over the last few years as it seeks for its sustainable development [2,3,5]. Nevertheless, some of its techniques are still being questioned [6]. The different points of view with regards to its costs and benefits are still to be reconciled from an economic, social, and environmental perspective [7].

On the one hand, aquaculture production plants are sustainable and cost effective from the producers’ point of view [8]. The production methods are increasingly efficient, with outputs 10 times greater than agriculture farms of similar dimensions [5]. For some regions, and mainly in some developing countries, it represents a substantial part of their business [4]. Aquaculture also has the capacity to alleviate the over-exploitation of some natural aquatic resources [8]. It can also represent a quality food supply for a growing human population [9,10], and the possibility of partial harvests [11].

Nevertheless, aquaculture can also pose some environmental challenges [12]. In fact, some of the chemical products used in aquaculture may end up reaching consumers or affecting food quality and/or its organoleptic characteristics [2,13,14,15]. Other environmental hazards, such as pollution [16], the spreading of non-native species or the alteration of natural habitats, should also be considered [17]. Finally, some conflicts of interests may appear with other local or regional agents competing for the same resources (some industries, traditional fishing, ports, or tourism) [18,19].

For this reason, it is necessary to carry out a study on the current social acceptance of this industry, in order to determine its current status with regards to its maximum social carrying capacity. This is a useful concept that should allow us to determine the actual acceptable limits for aquaculture expansion [1]. In this context, we could define the point of saturation of the sector as the level at which it exceeds such limits, and starts to be considered as a major concern from a social point of view [20]. This objective, which represents the main reason for our work, is still at a developing stage and, therefore, further progress is still required [21].

Society, in general, shows some concerns with regards to the economic, social, cultural, and environmental impact of aquaculture in those places where it takes place [22,23,24]. The sustainability of the sector requires environmental neutrality, as well as social acceptance [25]. Social acceptance is a very important aspect for this activity, and it has been studied in the literature from different approaches [7,10,26,27,28].

Firstly, from a socioeconomic standpoint, the development of aquaculture is socially accepted and economically feasible [29]. According to this approach, we should also remark the works by Little et al. that establish critical limits for social saturation and indicators that make it possible to measure its social carrying capacity, i.e., the developing stage where farm development gives place to relevant conflicts with other human activities/interests [30]. Some studies on seafood farming highlight its positive economic impact against its negative effect on the environment [31]. In particular cases, the support for aquaculture is mainly based on social rather than environmental reasons [32]. In this sense, many efforts have been made to develop the sector in a more sustainable manner, so that it is better accepted by the different groups of interest [23].

A number of works can be found in the literature that deal with aquaculture’s environmental issues by analysing different approaches, its sustainability, and the role that should be played by the corresponding public administrations in relation to these challenges [7,24,33,34,35,36,37]. Other studies emphasised the positive aspects associated to this industry’s environmental sustainability despite scarce social awareness [38,39,40].

Regarding the characteristics of the products obtained from this industry, they are usually considered cleaner than traditional fish, but in fact, it may contain some toxic substances [2]. Acceptance based on product labelling has also been analysed, and a better perception has been noticed when more complete information on sustainable procedures is provided [41]. Consumers would even be prepared to pay higher prices for seafood if higher safety food standards were guaranteed [28,42].

Finally, from an ethical point of view, fish welfare is also a concern [43,44], mainly for urban citizens and/or the younger sector of the population.

In this context, the main purpose of this work is to study the social acceptance of food obtained from aquaculture plants and compare it that of other products obtained from traditional fisheries. This study has focused on the population from the Andalusian Atlantic coast, in Southwest Spain and, by identifying the most critical population groups and their attitude towards this kind of food, should contribute to determine the social acceptable level for this sector and for its development before it reaches its point of saturation. For this purpose, we have conducted 579 face-to-face interviews, which have resulted in a representative social acceptance index that should cast some light on the future development of this production sector.

Our work could be considered as innovative, since, although Spain is the leading aquaculture producer in the European Union, not much research on the socio-economic carrying capacity of Spanish aquaculture industry has been carried out. According to the information published at Web of Science, we should highlight a proposal for a bio economic fish farm model in the Canary Islands [45]. Other works have studied this topic in a more indirectly way. For example, another case in the same islands focused mainly on its environmental impact [46]. There is also a study about mussels in Galicia [47]. In Andalusia, geographic information systems were used to improve environmental management and achieve social acceptability in Cadiz, among other considerations [48]. Since they did not focus on our topic as their primary goal, their methodology was very different, and the procedures employed should be rather associated to experimental fields.

We have focused our study on this geographic scope, because marine aquaculture emerged in this Atlantic area a century ago [49]. Moreover, Andalusia had approved 155 aquaculture sites in 2018, a large part of them (129) located inland [49]. The importance of the Atlantic Provinces lies in the fact that 131 of the Andalusian establishments are mainly located in them (84.51%), with a coverage of just 20.13% of the region surface. Together with the historical importance mentioned above, this area deserves a more detailed research like this one.

This paper will describe the methodology employed for this investigation. Then, the results from the surveys that have been carried out will be outlined. After that, the results will be discussed, and finally, our main conclusions based on those results will be elaborated.

## 2. Materials and Methods

As the main objective of this work, the social acceptance of aquaculture will be decisive in determining its “acceptable” level of development in these areas. The particular social carrying capacity in a specific area can be optimised, according to its general level of acceptance, and by identifying the demographic groups that are more reluctant to consume aquaculture products.

**Figure 1:**
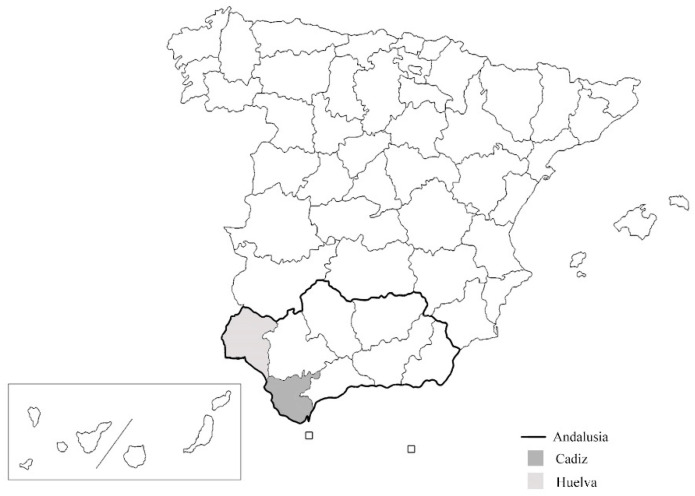
Andalusian Atlantic coast in Spain.

During the first stages of the study, we referred to other sources that would provide the theoretical framework for our main objective. The data obtained was used as a guideline to elaborate the subsequent questionnaire (see the Appendix A), with the use of primary sources through surveys. This is a relevant contribution of this work, since it supposes direct information from the users of the object of study. A market research company collected the survey data in the provinces of Cadiz and Huelva, the only two Atlantic provinces in Andalusia, as can be seen in Figure 1.

We opted for this methodology instead of social experiments for various reasons. First, we needed a larger, faster sample than we would get from social laboratory testing. Second, we have to meet the requirements from the project financing institution. Unfortunately, the use of experimental procedures would make the research more costly. Both approaches could be complementary and quite enriching, nevertheless, a greater budget would have to be allocated to fully cover both of them.

Our initial proposal included an objective scenario of 550 surveys and established a definite population sample. The polling company carried out the fieldwork during September 2018 by means of computer-assisted face-to-face surveys. The company completed the verification of at least 20% of the surveys delivered. This should guarantee that each individual had been actually interviewed. Likewise, they verified the consistency of the answers by asking some of the questions that were included in the survey again.

After leaving out the surveys that did not pass the researcher’s filter, we ended up with a total of 579 surveys. This represents a sampling error of +/- 4.16%, with a confidence level of 95.5%, assuming a maximum indeterminacy of around p = q = 0.5.

The polling company submitted a detailed report consisting on a simple statistical analysis of the variables, including the objective and quantifiable data. Then, we applied a number of advanced statistical methodologies to such data. As far as this work is concerned, we performed univariate analyses, mainly to determine position and dispersion measures, as well as frequency distributions. In addition, we carried out the necessary bivariate analyses to identify the possible dependencies between the variables (Pearson’s X^2^ tests, indicated as *).

After the revision of reference databases (such as Web of Science or Google Scholar), there is not an index like this in this field. We found, on the other hand, a good example of our methodology that had been applied to a paper on job insecurity in Toluca (Mexico) [50]. Both the method and the results were similar to what we pursue here. In our example, we selected these: “better quality”, “better taste”, “better price”, “more environmentally friendly”, “more employment”, “better employment”, “healthier”, “more variety”, and “improved availability”. We have worked with them as a semantic differential scale with 3 points, where (1) represents the greatest support for traditional fishing and (3) for aquaculture.

Before conducting a factor analysis, we validated this method by applying a Bartlett’s sphericity test [51]. This test allowed to contrast the null hypothesis that the correlation matrix is an identity, which indicated that the factor model is not suitable.

We estimated Cronbach’s alpha value in order to determine the internal consistency or reliability of these variables. Generally, any scores greater than 0.7 are acceptable [52]. We also conducted a Kaiser-Meyer-Olkin test, which measures how adequate the sample is [51]. Factor analysis would be appropriate when values remain between 0.5 and 1.

Afterwards, we carried out a factor analysis using Statgraphics Centurion XVII software. The type of factorisation applied was principal component analysis (PCA), with a varimax rotation. This type of PCA is the most commonly used and is suitable for a small number of components.

Based on a combination of two factors, the formula to determine aquaculture social acceptance index (SAI) would be as follows:

SAIj = V1 ∗ F1j + V2 ∗ F2j,(1)

where j = 1,… n, where n is the number of subjects observed;

F1j and F2j are factors 1 and 2, which correspond to the scores from each observation j;

V1 and V2 are each factor’s weighting, which is associated to the variability explained.

Finally, once SAI calculations were completed, we proceeded to stratify them. We applied the Dalenius-Hodges method [53] to determine the five strata. This method consists of forming several groups where internal variance is the smallest, while the variance between groups is the largest one. In other words, the groups obtained are as uniform as possible inside, but they are as heterogeneous as possible between them.

## 3. Results

Table 1 shows the distribution of the samples that had been collected.

Table 2 shows the respondents’ degree of satisfaction with aquaculture fish as a food. We can notice a high degree of satisfaction, since 44.74% indicate that they feel very or quite satisfied with this food, although 19.17% admit that they feel little or not satisfied at all.

When we look at the degree of satisfaction according to income levels, perception improves as this variable increases. In fact, for example, the percentage grows from 33.07% to 64.52% when adding the very and quite satisfied categories. On the other hand, the population group with monthly incomes at EUR 900 or less are the ones to position “not at all satisfied” (17.69%) more frequently. This percentage drops to 6.45% when respondents belong to the over EUR 2400 monthly income category.

The respondents’ opinion on the possibility of an increase or decrease of the aquaculture activities in their area over the next years in comparison to traditional fisheries was then analysed. It can be seen from the data in Table 3 that just over half the respondents seemed to be favourable to an increment of such activities in their region. Just nearly a tenth of them believed that this activity should be reduced over time. According to these results, aquaculture has not reached a saturation point in this geographical region from the social point of view. When results are considered according to respondent’s gender, it is noteworthy that men appear as more receptive to a prospective positive evolution of the sector, since up to 57.71% of them state that it should increase, while only 45.71% of women hold the same position (*p* = 0.0212*). No significant differences between the monthly income categories have been found.

The respondents’ vision on this aspect should be seen together with their overall perception on the advantages and disadvantages of aquaculture. Table 4 presents the results corresponding to the respondent’s view of aquaculture advantages and disadvantages. More than one-third of the population sample indicates that this sector is considered to improve the economy in the area, one-fourth of them consider that fish product prices are a positive aspect, and, finally, one-fifth of the respondents state that this industry can provide larger amounts and variety of fish throughout the whole year. There were only a few comments with regards to environmental concerns and the possibility of species recovery.

According to respondents’ gender, economic improvement and the amount of product available are the most important aspect for men (39.13% and 21.34%), while women consider these aspects less valuable (37.12% and 18.10%). In contrast, women 27.30% value the affordable price of aquaculture products slightly more than men do (25.30%). It should be noted that when the data is analysed according to the respondents’ income levels, those with the highest purchasing power are also the ones to value the most the potential of aquaculture to improve the economy in the area (54.84%) and to commercialise more affordable products (29.03%).

Table 4 also includes the data on aquaculture possible disadvantages. In this respect, it is worth mentioning that half the respondents state that this industry does not present any inconveniences, while about one-fifth of them point to the abusive use of industrial feeds and chemical products. Specifically, according to gender groups, 22.53% of men and 17.48% of women point toward this disadvantage. With regards to worse product quality and taste, 9.49% and 7.67% of men and women cite this as an issue, respectively, while its possible negative effect on traditional fishing scores at 7.51% and 4.91% (*p* = 0.0237*), respectively. It should also be noted that the population group with the highest income is the most concerned over its effect on the traditional fishing sector and over the potential consequences of abusing chemical products (*p* = 0.0298*).

Table 5 compares aquaculture against traditional fishing in order to establish an acceptance ranking for some related aspects. The respondents specifically indicate that aquaculture produces cheaper fish (70.47%) that is more easily available in supermarkets (67.70%). In contrast, traditional fishing provides a wider variety of greater quality. It is also healthier, tastier, and more environmentally friendly.

We can see that these responses could be characterised by a pattern similar to that observed for the previous questions. Thus, by gender, men appear to be more supportive towards aquaculture than women, who seem to be more hesitant about its advantages. For example, 74.70% of men consider its products as more affordable, while only 67.18% of women see this aspect as an advantage (*p* = 0.0436*). Furthermore, men perceive aquaculture to be more environmentally friendly (27.67%), when just 21.17% of women agree with this aspect. When the answers are analysed according to income population groups, we can perceive a positive relation between supportive attitudes and greater monthly income. For example, most of the respondents from all the monthly income categories consider that aquaculture can provide more affordable products, nevertheless, only 60% of the respondents from the EUR 900 monthly income group agree with this statement, while the figure goes up to 80.65% in the population category whose income is over EUR 2400 a month (*p* = 0.0092*). With regards to health aspects, the support for traditional fishing stands out in all the population categories but decreases from 83.85% to 64.52% as we move from lowest to highest income levels (*p* = 0.0006*). A similar thing happens when variety is evaluated, falling from 80.77% to 58.06% (*p* = 0.0001*) as income level goes up.

Table 6 shows the percentage of the respondents who consider that the public institutions provide support to each kind of fish producer. More than one-third of the sample population estimate that aquaculture activities are backed up twice as much as traditional fishing. The support by the public administrations is seen as higher among men (40.32%) than among women (30.98%) (*p* = 0.0076*). By income level, the perception that aquaculture receives more support increases as income does so, climbing from 27.69% until up to 51.61%, which corresponds to the highest income category (*p* = 0.0381*).

At this stage of the research, in order to determine the acceptance index, we must take into account some of the considerations that were previously considered for the initial PCA. Firstly, the Cronbach’s alpha for these items is 0.695, which is near the minimum value accepted to confirm its reliability [52]. Table 7 also shows that, according to the Kaiser-Meyer-Olkin index, the sample is adequate for the study [51]. Bartlett’s test results are also positive, and, therefore, the factorial analysis can be conducted.

Table 8 shows the factors before and after varimax rotation. Only two of those factors were selected, since the eigenvalues of the third one are lower than one (1). The two selected factors explain nearly half the data variability. Table 9 reflects the loads of the original variables for each factor obtained. In this way, factor 1 consists of the inherent characteristics of food associated with the variables “better quality”, “better taste”, “more environmentally friendly”, and “healthier”. On the other hand, factor 2 represents more external factors, such as those generated through the activities of the companies in the sector: "better price”, “more employment”, “better employment”, “more variety”, and “improved availability”.

In this way, we calculated the index of social acceptance (SAI) of aquaculture according to the following formula:

SAIj = 0.2682 ∗ F1j + 0.2183 ∗ F2j,(2)

The percentage of variance explained were 0.2682 and 0.2183. Then, the indices of the 579 samples were determined for the range from -56.29058 until 110.9008. In this case, the samples were divided into five levels following Dalenius and Hodges stratification procedure [53]: very low, low, medium, high, and very high.

Table 10 shows the frequency distribution that was obtained, where 32.12% of the samples indicate a high or very high acceptance of aquaculture activities, while acceptance would be low or very low for 42.14% of them.

**Figure 2:**
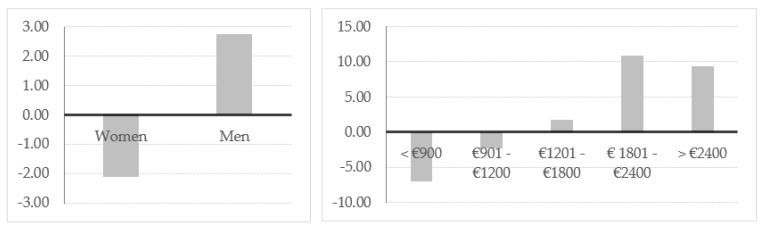
Index of acceptance by gender and income level.

As we can see in Figure 2, the average index obtained is higher for men than for women. With regards to the respondent’s income level, this indicator increases as the corresponding income category does, with negative or lower values corresponding to the lower income categories.

**Figure 3:**
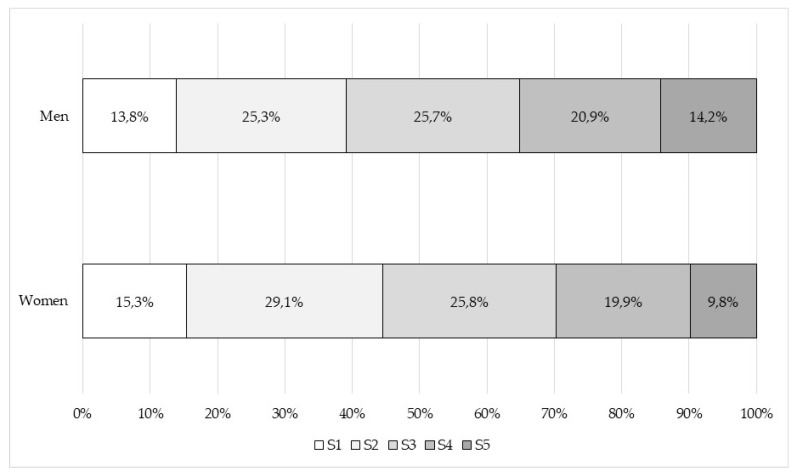
Social acceptance index (SAI) percentage distribution within gender categories.

Figure 3 shows the distribution of the SAI within each gender category. It can be seen that the percentage of men increases as the degree of acceptance goes up. Therefore, men show a high or very high acceptance level with a percentage of 35.2%, i.e., 5.4 percentage points above women’s level of acceptance (29.8%).

**Figure 4:**
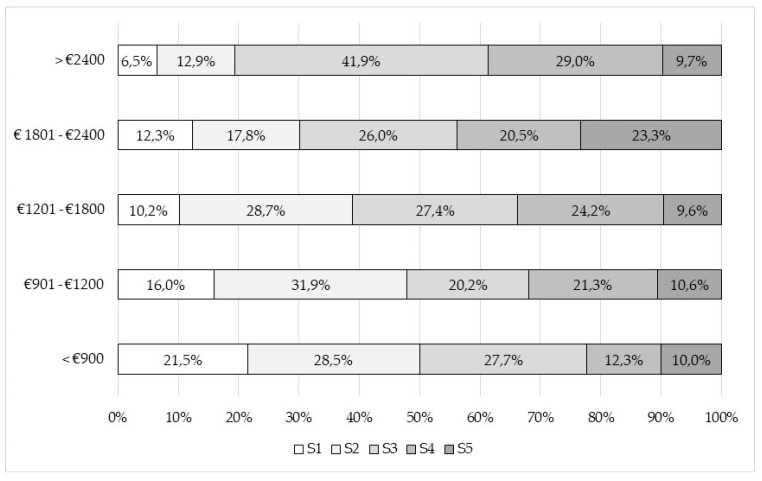
SAI distribution within income level categories.

Finally, Figure 4 shows the SAI distribution according to income level categories. In can be seen that SAI goes up as income does so. Thus, the population category with income below EUR 900 show an acceptance index of 50% (medium, high, or very high), while the respondents from the highest income category reach a SAI of up to 80.6%. In the same way, the lowest SAI levels correspond to the categories with higher incomes.

## 4. Discussion

The general objective of this work is to determine the social acceptance in Andalusian Atlantic provinces (Spain) of aquaculture industries compared to that of traditional fisheries based on their corresponding social carrying capacity. This data should contribute to the development of this industry by preventing unwanted changes in this geographical region. For this purpose, the index of social acceptance, as the representative perception of aquaculture by the population of interest, has been determined by reducing all the possible variables to just a few relevant factors.

Our study is justified on the basis of the scarce research works on Spanish aquaculture activities that can be found in the literature, despite being the leading producer in the European Union. Most works on this topic are still at a development stage, and, consequently, further studies are still required [21] to realise the necessary development of this industry.

The results from our study are consistent with those found in the literature. Thus, general respondents from these provinces showed a high level of satisfaction with aquaculture (44.74%) [54]. In fact, according to the general SAI that has been determined for the whole population sample, 32.12% of the respondents showed a high or very high acceptance level, even if these results are below the expected results when compared to previous observations.

In this order of things, most respondents believe that aquaculture activities should increase in the next few years (50.95%). In addition, more than one-third of the respondents (35.06%) think that official institutions largely provide support to this industry. As a result, this support from the administration might be perceived (or is sometimes perceived) as a threat to traditional fisheries [7].

Consumers seem to be aware of the considerable benefits that aquaculture represent with regards to employment and economic improvement in this area (38%) [25]. In this sense, the outcome of our study indicates that aquaculture if perceived as a source of more affordable food (26.42%), of a greater variety and amount (19.52%). This is in agreement with the conclusions reached by Claret et al. [55]. On the other hand, regarding the possible drawbacks, it should be mentioned that over half the population sample (54.58%) did not point out any specific negative aspects. Nevertheless, some of the respondents (19.69%) stated that abusive use of feeds and/or chemicals should be avoided by the operating companies, while the quality and taste of the fish should be improved (8.46%). Previous studies had already highlighted the environmental problems and health risks related to salmon-producing activities [1]. On the other hand, some works claim that consumers would be prepared to accept higher prices if a greater degree of food safety was ensured [2,42]. It seems rather obvious that consumers’ perception should be taken into account for the development of any prospective studies on the technical and commercial aspects of this industry.

The results obtained from our survey have been analysed according to gender and income level categories. In this regards, we would like to point out that fish consumption has already been studied from these perspectives, while acceptance of aquaculture in itself has never been covered by any of the studies that can be found in the literature until present. This is why we regard our work as a genuinely innovative approach.

First of all, some relevant differences can be highlighted between men and women’s perception of the subject. While men have a more positive attitude towards aquaculture, women seem to stand on a more reserved position. For example, men consider it more environmentally friendly than women do.

The average acceptance index obtained is higher for men than it is for women. In fact, men’s general acceptance index is 5.4 percentage points greater. Particularly, with regards to a prospective future increment of this activity, men are more favourable than women by 12 percentage points. Additionally, the percentage of men who perceive that public administrations provide support to this industry is 9.3 points higher compared to that of women.

Moreover, men grant higher scores than women to other aspects, such as benefits, economic improvement, and product quality. In contrast, women perceive more affordable prices as a more relevant aspect than men do. Regarding the respondents’ perception of aquaculture’s negative aspects, men score higher than women when it comes to pointing out the abuse of artificial feeds and/or chemicals, lower product quality, and taste as the possible disadvantages when compared to traditional fishing.

Some substantial differences can also be found with regard to the different monthly income categories. In general, the degree of support for aquaculture increases according to higher income levels. For example, the total percentage of respondents that are very or quite satisfied with aquaculture doubles as we move from the EUR 900 monthly income category to the over EUR 2400 monthly income level. In the same way, the index of social acceptance increases according to income categories, where lower income groups also show the lowest acceptance levels. Thus, this indicator reaches a difference of 30.6 percentage points between the lowest and the highest income categories.

When questioned on aquaculture advantages and disadvantages, those respondents from the higher income categories showed more concern in relation to economic improvements and affordable prices, but also about the possible impact on traditional fishing, as well as the negative consequences of abusive employment of chemical products. Aquaculture products are perceived as more affordable by all the population income categories, although support goes up in direct relation with greater incomes, with a difference of 20.65 percentage points between the lowest and the highest categories. On the other hand, the percentage of preference for traditional fishing products decreases as income increases. For example, the percentage of respondents that perceive traditional fish as healthier falls by 19.33 percentage points between the lowest and the highest income population categories. A similar thing happens with the perception of product variety, which equally drops by 22.71 per cent as income goes up.

In relation to the prospective evolution of this sector, most population categories seemed to perceive aquaculture as a growing industry. Likewise, aquaculture is also perceived as an industry that receives support from official institutions to a greater extent.

Since the most reluctant groups (women and low-income population) are key collectives for the development of the aquaculture industry. Communication campaigns (informative or advertising) should target these profiles to spread the benefits of these products in order to reduce any concerns about them [22,25,55]. In this sense, social acceptance by this sector should be gained by enhancing the quality and sustainable aspects of aquaculture food products [25]. Promotion campaigns should focus on spreading the idea that they take into account consumers ‘opinion and pay special attention to any specific information in relation to their concerns [41]. These targets should be more easily achieved when related to aquaculture’s positive aspects, even if its more obviously negative sides would be a harder obstacle to overcome.

We can, therefore, affirm that according to the results obtained, aquaculture is far from its saturation point expressed as its maximum social carrying capacity. Analogous studies based on other Spanish regions (Galicia) have come to a similar conclusion [47].

## 5. Conclusions

First of all, we would like to highlight that, according to our results, there is a considerably positive attitude towards aquaculture products in general. Nevertheless, we should also point out that our expectations have not been fully met in this regard. In this sense, it is noteworthy that the two dimensions that have been brought to light by the resulting index should be considered as intrinsically related both to the product as well as to the activity of the companies. In addition, the fact that most people think that this activity should increase in their geographical region seem to indicate that this sector is socially perceived as far from its saturation point.

Based on the behavioural profiles that have been obtained, we can conclude that the attitude towards aquaculture is slightly better among men than among women, who prefer the products from traditional fisheries. A more positive attitude has also been observed in a direct relation with higher income levels of the population. Accordingly, the respondents with lower income tend to stand at a more reluctant position with respect to this industry. Consequently, we would recommend that any media campaigns that intend to increase the current acceptance levels of these industrial activities should mainly target the most critical population categories that have been identified by our research work [32,35].

We should finally highlight some of the peculiarities and limitations that we consider should be more thoroughly analysed in future studies on this topic. In the first place, the emphasis on socio-economic aspects leads the authors to apply a survey methodology that is more typical of social sciences, other than experimentation, which is more prevalent in other fields of knowledge.

Undoubtedly, in coming years, as further lines of research are covered, we will apply more complex and advanced techniques that will enrich these conclusions, even by combining them with experiments in social laboratories. According to the index obtained, these kinds of indicators have always a risk of excessive simplification [21]. We can develop them more deeply in the future. We should also delve into aspects such as the acceptance analysis by the age of the respondents, because we rejected this question after receiving the surveys. Furthermore, a joint analysis of the survey results according to gender and income level is still to be carried out. For these variables (gender, income level, age) we could apply a qualitative methodology to identify those factors (maybe society, family, culture, or education) that determine these opinions, and the possible existence of cognitive biases.

Furthermore, in future approaches, we would like to broaden this analysis in order to include other Spanish geographic areas or to focus on particular aquaculture species. We would also consider the possibility of applying similar studies to other countries, since their differences in consumption habits and their particular experience in a diversity of contexts might be rather enlightening.

## Figures and Tables

**Table 1:** Sample distribution.

Gender	n	%	Income Level (€/month)	n	%
Women	326	56.30%	Less than or equal to 900	130	22.45%
Men	253	43.70%	901–1200	188	32.47%
			1201–1800	157	27.12%
			1801–2400	73	12.61%
			More than 2400	31	5.35%
TOTAL	579	100%	TOTAL	579	100%

**Table 2:** Degree of satisfaction with aquaculture fish as food.

	n	%
Very satisfied	54	9.33
Quite satisfied	205	35.41
Somewhat satisfied	133	22.97
Little satisfied	51	8.81
Not at all satisfied	60	10.36
Don’t know/No answer	76	13.13
TOTAL	579	100

**Table 3:** In the next coming years, aquaculture should.

	n	%
Increase	295	50.95
Remain the same	158	27.29
Decrease	51	8.81
Don’t know/No answer	75	12.95
TOTAL	579	100

**Table 4:** Perception of the potential advantages and disadvantages of aquaculture.

Advantages	n	%	Disadvantages	n	%
Economic improvement (more employment)	220	38.00	Abuse of chemicals	114	19.69
Cheaper price	153	26.42	Worse quality and taste	49	8.46
Greater amount and fish variety	113	19.52	Damage to traditional fishing	35	6.04
Better quality, healthier	77	13.30	Environmental impact	11	1.90
Respect for the environment; recovery of species	17	2.94			
Others	13	2.25	Others	17	2.94
None	62	10.71	None	316	54.58
Don’t know/No answer	48	8.29	Don’t know/No answer	71	12.26
TOTAL	703	121.43	TOTAL	603	105.87

**Table 5:** Comparison between aquaculture fish and traditional fish.

	Aquaculture Fish		Don’t Know/ No Answer		Traditional Fish	
	n	%	n	%	n	%
Better price	408	70.47	72	12.44	99	17.10
Improved availability	392	67.70	93	16.06	94	16.23
More employment	242	41.8	80	13.82	257	44.39
Better employment	238	41.11	117	20.21	224	38.69
More environmentally friendly	139	24.01	100	17.27	340	58.72
More variety	123	21.24	55	9.50	401	69.26
Healthier	82	14.16	51	8.81	446	77.03
Better quality	54	9.33	41	7.08	484	83.59
Better taste	30	5.18	55	9.50	494	85.32

**Table 6:** Perception of support by public administrations.

	n	%
Traditional fish	101	17.44
Aquaculture fish	203	35.06
Both equally	57	9.84
None	106	18.31
Don’t know/No answer	112	19.34
TOTAL	579	100

**Table 7:** Previous principal component analysis (PCA) results.

Test	Value
Kaiser-Meyer-Olkin index	0.74866
Determinant of the correlation matrix	0.175141
X^2^	1000.2913
Degrees of freedom	36
*p*	0.0000

**Table 8:** Variance explained.

	Original Values			Values after Varimax Rotation	
	Factor 1	Factor 2	Factor 3 ^1^	Factor 1	Factor 2
Eigenvalues	2.7741	1.6043	0.9521	2. 4138	1.9646
% Variance	30.82%	17.83%	10.58%	26.82%	21.83%
% Accumulated Variance	30.82%	48.65%	59.23%	26.82%	48.65%

^1^ Not Selected.

**Table 9:** Factor loads after Varimax rotation.

	Factor 1	Factor 2
Better quality	0.8387	0.0096
Better taste	0.7695	−0.0040
More environmentally friendly	0.5134	0.2393
Healthier	0.8076	0.1366
Better price	−0.0730	0.6552
More employment	0.2386	0.6437
Better employment	0.2568	0.6362
More variety	0.2529	0.4061
Improved availability	−0.1015	0.6894

**Table 10:** Stratification of the acceptance levels.

Categories	Stratum	Square Root of Absolute Cumulative Frequency (Intervals)	Acceptance (Intervals)	n	%
Very low	S1	0; 28.6522	−56.2906; −32.8838	85	14.68%
Low	S2	28.6522; 57.3044	−32.8838; −9.4770	159	27.46%
Medium	S3	57.3044; 85.9567	−9.4770; 10.5860	149	25.73%
High	S4	85.9567; 114.6089	10.5860; 40.6804	118	20.38%
Very high	S5	114.6089; 143.2611	40.6804; 110.9008	68	11.74%

## Appendix A

Questionnaire used for the survey (Extract)

**What is Your Level of Satisfaction with Aquaculture Fish?**					
Not at all satisfied	Little satisfied	Somewhat satisfied	Quite satisfied	Very satisfied	Don’t know/No answer

**According to You, Which Advantages Can Aquaculture Companies Generate in This Region? (You Can Select Several Options)**
Economic improvement (more employment)
Greater quantity and variety of fish
Better quality, healthier
Cheaper prices
Environmental care; recovery of species
Others
Don’t know/No answer

**According to You, What Disadvantages Problems Can Aquaculture Companies Cause in This Region? (You Can Select Several Options)**
Abuse of chemicals
Impact on the environment
Worse quality and taste
Damage to traditional fishing
Others
Don’t know/No answer

**In the Coming Years, Aquaculture in Your Region Should....**			
Increase	Remain the same	Decrease	Don’t know/No answer

**In Your Opinion, What Type of Fish Production are the Public Administrations Supporting the Most?**				
Traditional fisheries	Aquaculture	Both equally	None	Don’t know/No answer

**When Comparing Aquaculture and Traditional Fisheries, Which One do You Think Stands Out in These Aspects?**			
	Traditional	Don’t know/No answer	Aquaculture
Better price	○	○	○
Improved availability	○	○	○
More variety	○	○	○
Better quality	○	○	○
Healthier	○	○	○
Better taste	○	○	○
More employment	○	○	○
Better employment	○	○	○
More environmentally friendly	○	○	○

**Gender of the Respondent**	
Female	Male

**Monthly Gross Income Level of the Respondent (€)**				
900 or less	901–1200	1201–1800	1801–2400	2400 or over
